# Comparative Transcriptome Analysis of Two Peach Rootstocks Uncovers the Effect of Gene Differential Expression on Nitrogen Use Efficiency

**DOI:** 10.3390/ijms231911144

**Published:** 2022-09-22

**Authors:** Qiuju Chen, Min Lian, Jian Guo, Binbin Zhang, Sankui Yang, Kexin Huang, Futian Peng, Yuansong Xiao

**Affiliations:** State Key Laboratory of Crop Biology, College of Horticulture Science and Engineering, Shandong Agricultural University, Tai’an 271000, China

**Keywords:** peach, nitrogen use efficiency, comparative transcriptomics, *PpNRT2.1* gene, expression pattern, nitrate ion

## Abstract

Nitrogen is an important nutrient element that limits plant growth and yield formation, but excessive nitrogen has negative effects on plants and the environment. It is important to reveal the molecular mechanism of high NUE (nitrogen use efficiency) for breeding peach rootstock and variety with high NUE. In this study, two peach rootstocks, Shannong–1 (S) and Maotao (M), with different NUE were used as materials and treated with 0.1 mM KNO_3_ for transcriptome sequencing together with the control group. From the results of comparison between groups, we found that the two rootstocks had different responses to KNO_3_, and 2151 (KCL_S vs. KCL_M), 327 (KNO_3__S vs. KCL_S), 2200 (KNO_3__S vs. KNO_3__M) and 146 (KNO_3__M vs. KCL_M) differentially expressed genes (DEGs) were identified, respectively, which included multiple transcription factor families. These DEGs were enriched in many biological processes and signal transduction pathways, including nitrogen metabolism and plant hormone signal transduction. The function of *PpNRT2.1*, which showed up-regulated expression under KNO_3_ treatment, was verified by heterologous expression in Arabidopsis. The plant height, SPAD (soil and plant analyzer development) of leaf and primary root length of the transgenic plants were increased compared with those of WT, indicating the roles of *PpNRT2.1* in nitrogen metabolism. The study uncovered for the first time the different molecular regulatory pathways involved in nitrogen metabolism between two peach rootstocks and provided gene reserve for studying the molecular mechanism of nitrogen metabolism and theoretical basis for screening peach rootstock or variety with high NUE.

## 1. Introduction

As one of the essential nutrition elements in the process of plant growth and development, nitrogen contributes 40–50% to the final yield of plants, and is an important component of proteins, nucleic acids and phospholipids in plants. Nitrogen use efficiency (NUE) refers to the efficiency of nitrogen fertilizer utilization by plants [1]. In general, the higher the NUE, the better the yield, and therefore, the application of nitrogen fertilizer is one of the routine ways to obtain high yields. However, due to the soil condition and plants, nitrogen fertilizer can never be 100% used [2,3,4], and nearly 50–70% of the nitrogen fertilizer applied to the soil cannot be used by plants [5]. Excessive nitrogen fertilizers not only adversely affect the yield and quality of plants but also causes pollution and damage to the environment and ecosystems. Under the same environment, the gene expression affects the phenotype of plants. Plants with different genetic backgrounds have different NUE. Therefore, it is important to strengthen research on the molecular mechanism of high NUE, which will provide guidance for breeding high NUE cultivars.

The forms of nitrogen absorbed by plants from the soil are NO_3_^−^-N and NH_4_^+^-N, and they mainly enter the cells from extracellular through ion transporters on the root cell membrane [6]. The protein family responsible for NO_3_^−^-N uptake is NRT protein family [7], and the protein family responsible for NH_4_^+^-N is AMT protein family [8]. At present, two types of nitrate absorption systems have been found in plants, one is low-affinity transport system (LATS), which uptake NO_3_^−^ from the soil when the nitrate is plentiful (>1 mM), and the responsible protein family is NRT1. Another absorption system is the high-affinity transport system (HATS), which plays roles when the external nitrate concentration is low (1 μM–1 mM), and the responsible protein family is NRT2 [9,10,11]. These two systems work together to ensure that plants can use a wide range of nitrate concentrations.

In Arabidopsis, 53 genes encoding NRT1 are cloned, of which 51 are tissue-specifically expressed [11], and *AtNRT1.1* induced by nitrate is a dual-affinity transporter that participates in low- and high-affinity transport systems through protein phosphorylation and dephosphorylation [12]. For the NRT2 gene family, most members are expressed in roots, and *AtNRT2.1* is the most studied [13,14,15,16]; in the gene deletion mutant, the high-affinity nitrate transport capacity is reduced by 75% [17]. Nitrate entering the assimilation pathway is reduced to ammonia by the catalysis of nitrate reductase (NR) and nitrite reductase (NiR), and then finally form various amino acids such as glutamic acid under the action of glutamine synthase (GS1 and GS2) to participate in the synthesis of biological macromolecules such as proteins [18]. Besides the nitrate transporter genes, there are many other factors involved in the nitrogen metabolism. For example, the synthetic precursor of ethylene, ACC, can reduce the expression of *BnNRT2.1* and nitrogen absorption [19], under low nitrogen condition, there is a feedback regulation between ethylene and *AtNRT2.1* [20]. TaNAC2-5A can bind the promoter of *TaNRT2.1-6B*, *TaNPF7.1-6D* and *TaGS2-2A*, and enhance grain yield and high nitrogen accumulation [21], *OsNPF6.1* can be trans-activated by OsNAC42 [22].

Peach tree, the third largest deciduous fruit tree in China, is a type of NO_3_^−^-N-loving plant, absorbs NO_3_^−^-N as the main nitrogen source, and has low- and high-affinity transport systems to absorb NO_3_^−^-N fertilizer [23]. Due to the lack of understanding of the nitrogen absorption and metabolism in peach trees, the phenomenon of excessive application of nitrogen fertilizers in peach production is relatively common, which not only leads to the imbalance of phosphorus, potassium and micronutrients, but also increases the workload of tree management, reduces fruit quality, and even causes physical diseases. In recent years, although researchers have made significant efforts to improve nitrogen utilization and established some standards and norms, and some achievements have been obtained in the research on improving NUE of fruit trees by changing the fertilization method and fertilization time [24,25], the understanding of nitrogen absorption and utilization of peach trees is still not in-depth and comprehensive. Compared with the herbaceous plants such as rice, *Brassica napus* and Arabidopsis, there are few studies on the molecular mechanism of nitrogen metabolism and breeding high NUE cultivars or rootstocks by using NUE genes.

In this study, four cDNA libraries of two peach rootstocks, Shannong–1and Maotao, with different NUE, treated with 0.1 mM KNO_3_ and KCL respectively, were constructed by high-throughput sequencing. Through comparative analysis between libraries and functional annotation of differentially expressed genes, it was found that the DEGs were involved in multiple growth and development pathways, including nitrogen metabolism, hormone metabolism and transcriptional regulation. The differentially expressed gene *PpNRT2.1* was overexpressed in Arabidopsis, and the transgenic lines showed a phenotype of higher plant height and chlorophyll content, and longer primary root length under low concentration nitrogen treatment, which was consistent with the previous related studies. For the first time, this study revealed the reason for the different NUE between the two peach rootstocks at the molecular level and provided a theoretical basis for studying the molecular mechanism of nitrogen metabolism in peach trees and breeding varieties and rootstocks with high NUE.

## 2. Results

### 2.1. The Different NUE between Shannong–1 and Maotao

**Figure 1:**
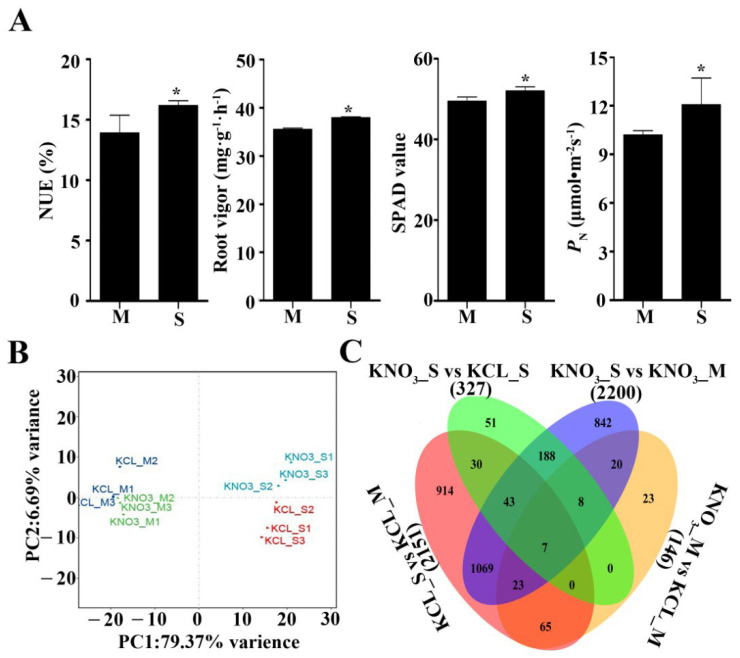
Analyses of nitrogen-related indices and comparison of four transcriptome libraries. (**A**) Analysis of NUE, root vigor, SPAD and *P*_N_ of Shannong–1 and Maotao. Error bars show the standard error between three biological replicates (*n* = 3). * significance at *p* < 0.05 according to two-tailed Student’s *t*-test. S: Shannong–1, M: Maotao. (**B**) Principal component analysis of four transcriptomes. M1, M2 and M3: Three biological replicates of Maotao roots. S1, S2 and S3: Three biological replicates of Shannong–1 roots. KCL and KNO_3_: the two rootstocks were treated by 0.1 mM KCL and KNO_3_ for 0.5 h. (**C**) Venn diagram analysis of all induced unigenes.

NUE is the degree to which nitrogen fertilizer is used in the season. Under the same growth condition, NUE is determined by the genetic background of plants and can reflect the nitrogen utilization ability of plants. Here, two peach rootstocks, Shannong–1 (S) and Maotao (M), were used as materials to measure their NUE, and result showed that the NUE of S was 17.16% higher than that of M, showing significant difference (Figure 1A). In addition, we also measured the NUE-related physiological indicators of root vigor, leaf SPAD and net photosynthetic rate of the two rootstocks, the indicator values of S were 6.81%, 5.20% and 18.24% higher than that of M, respectively (Figure 1A). The results reflected the NUE of S was higher than that of M.

### 2.2. Transcriptome Sequence Analysis of Shannong–1 and Maotao

To explore the reasons for the different NUE of S and M at molecular level, four cDNA libraries were constructed from the roots of the two rootstocks treated with 0.1 mM KNO_3_ and 0.1 mM KCL for 0.5 h by high-throughput sequence. After removing adaptor and low-quality sequences, 45.91 M (KCL_S), 47.71 M (KCL_M), 46.66 M (KNO_3__S), and 48.02 M (KNO_3__M) clean reads were obtained with Q30 > 93.18%, 93.31%, 92.26%, and 93.92%, respectively. Principal component analysis (PCA) showed good repeatability among the three biological replicates and the greater effect of 0.1 mM KNO_3_ treatment on S than that M (PC2) (Figure 1B). Through comparison analysis between libraries, we got more differentially expressed genes (DEGs) between the two rootstocks (2151 (KCL_S vs. KCL_M) and 2200 (KNO_3__S vs. KNO_3__M)) than that between the treatment group and the control group of the same rootstock (327 (KNO_3__S vs. KCL_S) and 146 (KNO_3__M vs. KCL_M)) (Figure 1C and Table S1 in Supplementary Materials).

**Figure 2:**
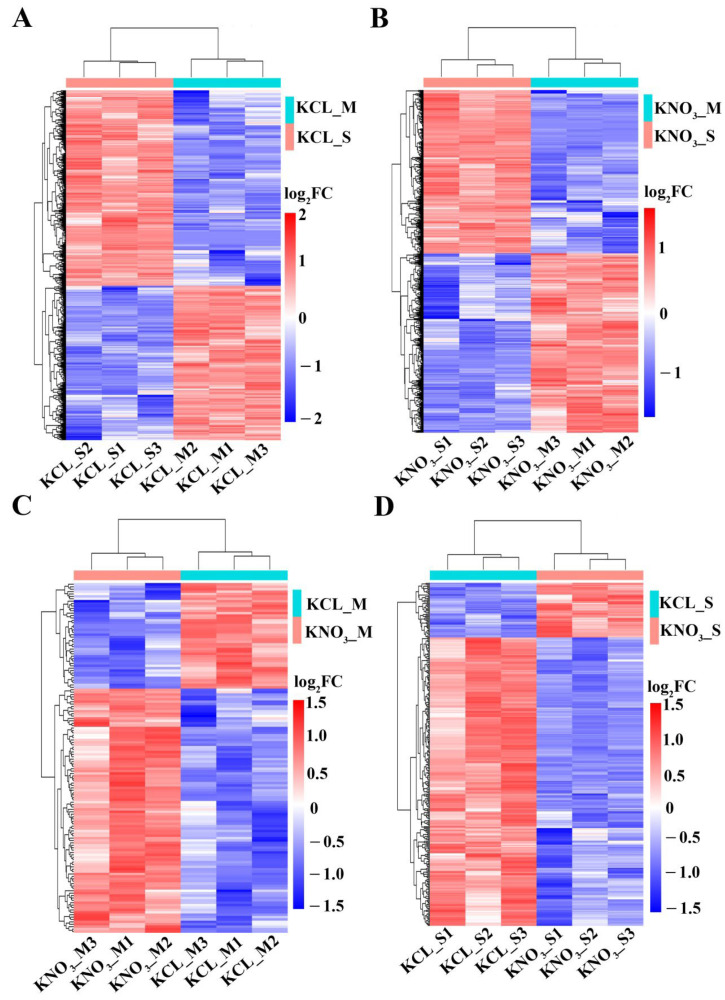
Clustering analysis of the differentially expressed genes. (**A**) Clustering analysis of DEGs identified from comparison group of S and M treated with KCL. (**B**) Clustering analysis of DEGs identified from comparison group of S and M treated with KNO_3_. (**C**) Clustering analysis of DEGs identified from comparison group of M treated with KNO_3_ and KCL. (**D**) Clustering analysis of DEGs identified from comparison group of S treated with KNO_3_ and KCL.

In addition, the whole expression profiles of the DEGs were presented in Figure 2. In the 0.1 mM KCL treatment group, compared with the expression level of genes in M, the number of up-regulated DEGs was more than the number of down-regulated DEGs in S. While in the 0.1 mM KNO_3_ treatment group, the number of up-regulated DEGs was less than the number of down-regulated DEGs in S (Figure 2A,B). For the same rootstock treated by 0.1 mM KNO_3_, the number of up-regulated DEGs was more than the number of down-regulated DEGs in M, while the number of down-regulated DEGs in S was more than the number of up-regulated DEGs (Figure 2C,D). These suggested that the differences in the number of DEGs identified were mainly driven by the distinct genetic backgrounds of the two rootstocks, and the gene expression patterns caused by KNO_3_ in the two rootstocks were different, maybe it was the reason that the two rootstocks had different responses to low concentration of KNO_3_.

### 2.3. Function Annotation for the DEGs

**Figure 3:**
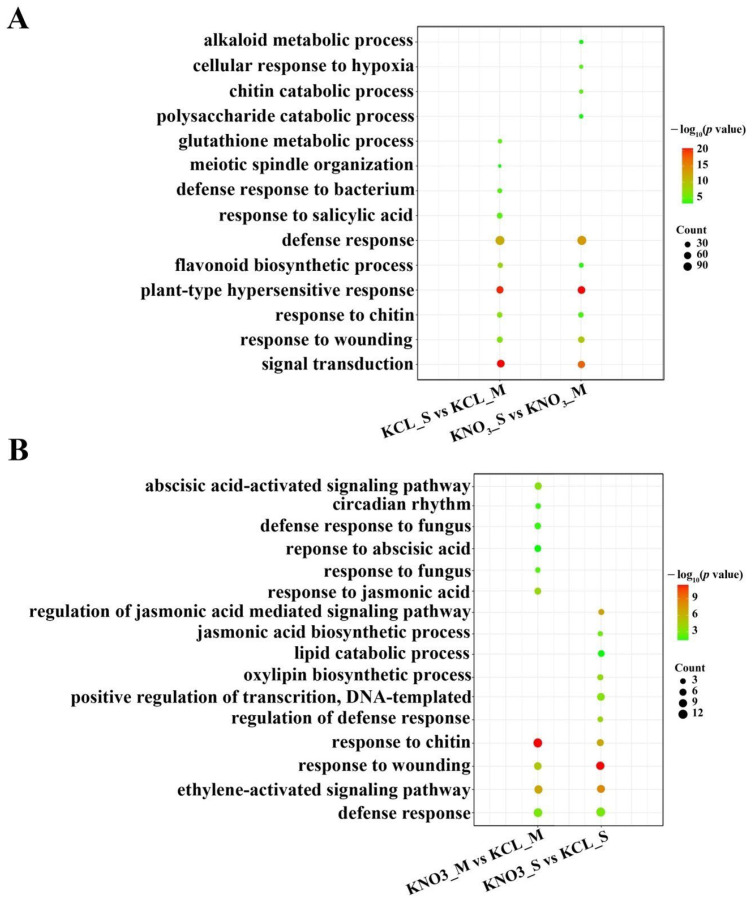
Gene Ontology (GO) enrichment analysis. (**A**) Enriched GO terms are common and unique to S versus M treated by KCL (KCL_S vs. KCL_M) and S versus M treated by KNO_3_ (KNO_3__S vs. KNO_3__M). (**B**) Enriched GO terms are common and unique to S treated by KNO_3_ versus S treated KCL (KNO_3__S vs. KCL_S) and M treated by KNO_3_ versus M treated by KCL (KNO_3__M vs. KCL_M). The size of the circle represents the number of DEGs in that GO term category, and the color of the circle indicates the −log_10_ (*p* value).

Gene ontology (GO) categories were performed to identify the major pathways underlying the observed differences between the rootstocks and treatment groups. Figures S1 and S2 in Supplementary Materials summarized the categorization of DEGs according to biological process, cellular component and molecular function. In order to identify the differences among the treatment groups, we carried out further comparative analyses based on biological processes. First, we identified GO terms that were shared between S and M samples. It could be seen that GO terms commonly enriched between control (KCL_S vs. KCL_M) and KNO_3_ treatment (KNO_3__S vs. KNO_3__M) comparison groups were “plant-type hypersensitive response”, “signal transduction”, “defense response”, “flavonoid biosynthetic process” and so on. The DEGs between S and M samples treated by KNO_3_ (KNO_3__S vs. KNO_3__M) were enriched in GO terms including biological macromolecular metabolism like “alkaloid metabolic process”, “chitin catabolic process” and “polysaccharide catabolic process” (Figure 3A). This suggested that different biological processes were affected by low-concentration KNO_3_. 

To understand the effect of low-concentration KNO_3_ on the two rootstocks respectively, we also identified GO terms that enriched in S response to low-concentration KNO_3_ comparison group and M response to low-concentration KNO_3_ comparison group (KNO_3__S vs. KCL_S and KNO_3__M vs. KCL_M). We found some categories of GO terms enriched in both comparison groups, such as “response to chitin” and “ethylene-activated signaling pathway”. In addition, each comparison group had its own GO categories. For example, after KNO_3_ treatment, “regulation of jasmonic acid mediated signaling pathway”, “oxylipin biosynthetic process” and “transcription” enriched in S comparison group, but not in M comparison group. The GO categories that were related to fungus and abscisic acid process, “response to fungus” and “abscisic acid-activated signaling pathway”, only enriched in M comparison group (Figure 3B). The results further showed the two rootstocks responded differently to low concentration of KNO_3_.

**Figure 4:**
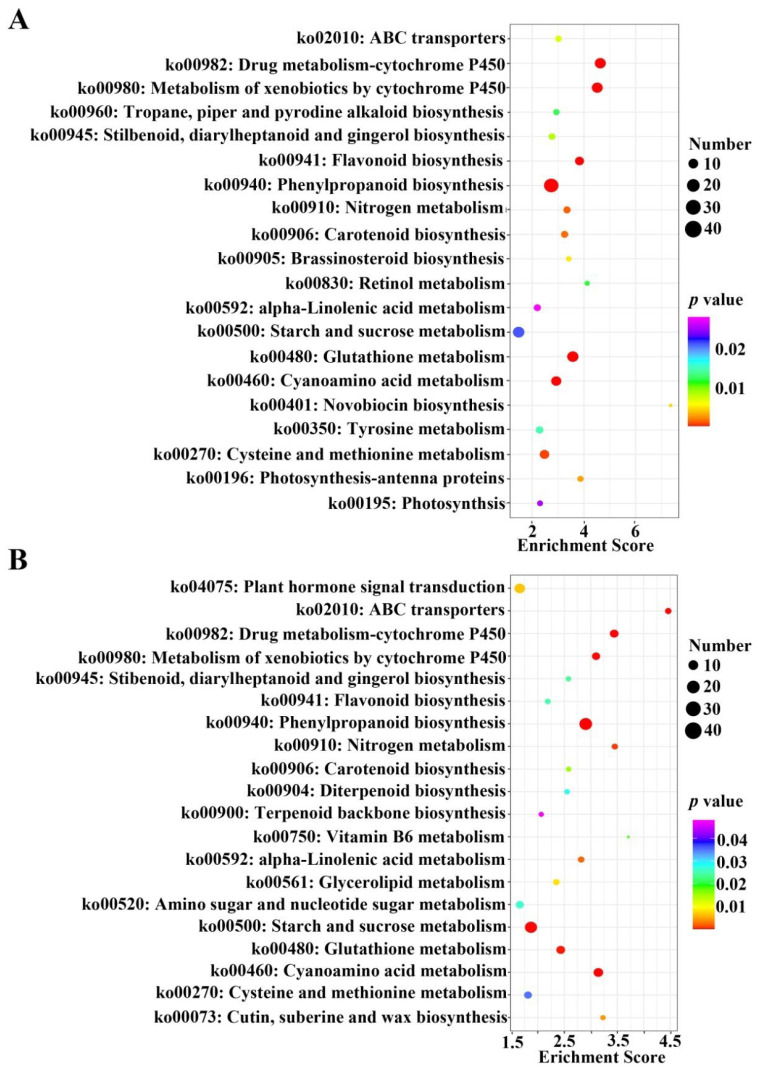
KEGG pathway enrichment analysis of significantly up- and down-regulated DEGs. (**A**) Enriched KEGG pathways of DEGs identified from S versus M treated by KCL (KCL_S vs. KCL_M). (**B**) Enriched KEGG pathways of DEGs identified from S versus M treated by KNO_3_ (KNO_3__S vs. KNO_3__M). The size of the circle represents the number of DEGs in that KEGG pathway, and the color of the circle indicated *p* value.

The KEGG pathway annotation for all DEGs identified from the two rootstocks was obtained as shown in Figure 4. In the comparison between S and M treated by KCL (KCL_S vs. KCL_M) and KNO_3_ (KNO_3__S vs. KNO_3__M), there were 20 significantly pathways enriched, respectively. From the results, we could see that after KNO_3_ treatment, some KEGG pathways were decreased in control comparison group (KCL_S vs. KCL_M), such as “Retinol metabolism (ko00830)”, “Novobiocin biosynthesis (ko00401)” etc. (Figure 4A), and some KEGG pathways were increased in KNO_3_ treatment comparison group (KNO_3__S vs. KNO_3__M), such as “plant hormone signal transduction (ko04075)”, “amino sugar and nucleotide sugar metabolism (ko00520)”, etc. (Figure 4B), and the *p* value of DEGs enriched in some KEGG pathways were decreased, such as “starch and sucrose metabolism (ko00500)”, etc. (Figure 4). The results indicated that these KEGG pathways and mechanism might play different regulatory roles in the process of nitrogen metabolism.

### 2.4. Analysis of DEGs Involved in Nitrogen Metabolism of Peach Rootstocks

**Figure 5:**
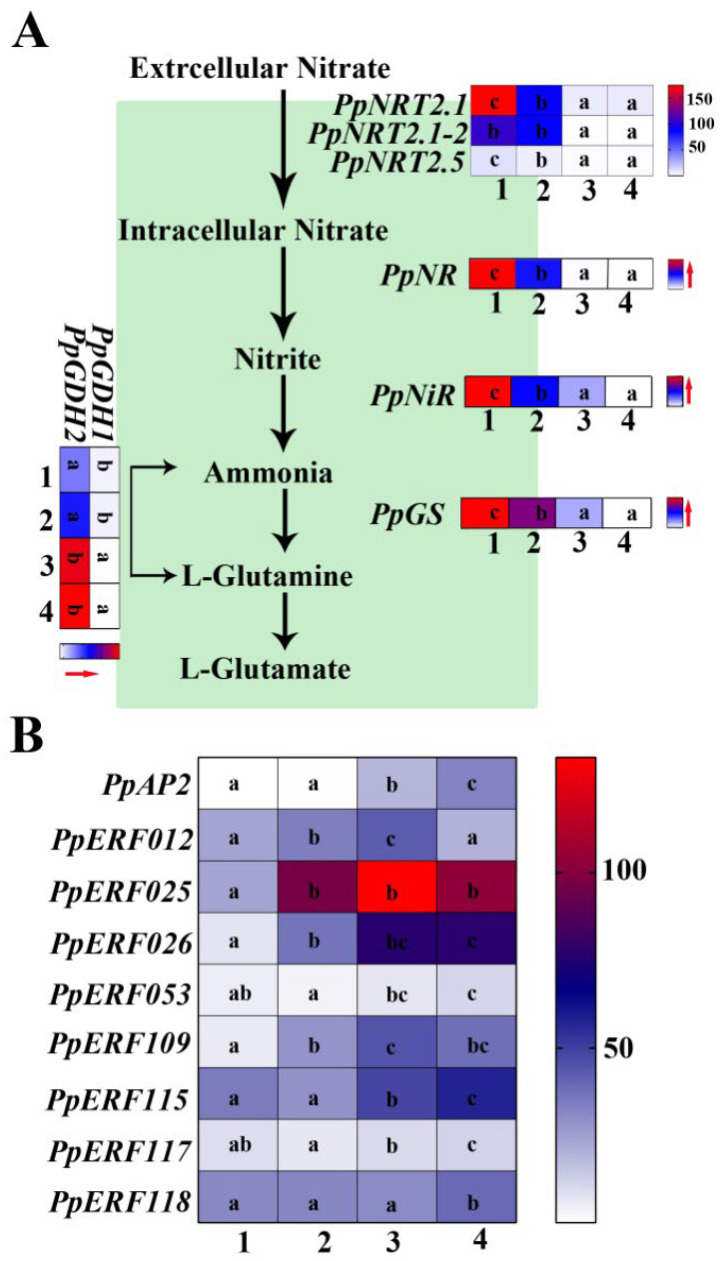
Nitrogen metabolic pathway in peach root. (**A**) Nitrogen metabolic pathway in peach root. The green background box represents the intracellular. *PpNRT2.1* (LOC18773076), *PpNRT2.1-2* (LOC18773670) and *PpNRT2.5* (LOC18793381): genes encoding nitrate transporters; *PpNR* (LOC18790070): gene encoding nitrate reductase; *PpNiR* (LOC18767486): gene encoding ferredoxin-nitrite reductase; *PpGS* (LOC18790849): gene encoding glutamine synthetase; *PpGDH1* (LOC18770115): gene encoding glutamate dehydrogenase 1 and *PpGDH2* (LOC18785018): gene encoding glutamate dehydrogenase 2. 1, 2, 3 and 4 represents KNO_3__S, KCL_S, KNO_3__M and KCL_M. The red arrow represents the increase of FPKM value. (**B**) The normalized expression level (FPKM) of some differentially expressed *ERF* gene family members in the four cDNA libraries analyzed by heat map. Different lowercase letters indicate significant differences at *p* < 0.05 by one-way ANOVA with Duncan (D)’s multiple comparisons test.

By screening KCL_S vs. KCL_M and KNO3_S vs. KNO3_M transcriptome data with *p* < 0.05, and then based on GO and KEGG annotation, the DEGs involved in nitrogen metabolism pathways were finally obtained, including *NRT2.1* family genes (*PpNRT2.1*, *PpNRT2.1-1*, *PpNRT2.5*), which play a major role in high-affinity nitrogen transport system, nitrate reductase gene (*PpNR*), nitrite reductase gene (*PpNiR*), and glutamine synthetase gene (*PpGS*). To facilitate the comparison and visualization of the changed transcripts in both rootstocks under low concentration KNO_3_, the nitrogen metabolite and transcripts normalized as FPKM (fragments per kilobase of exon model per million mapped fragments) were depicted in Figure 5A. It could be seen that the expression levels of these genes involved in nitrogen metabolism were significantly higher in S than in M, and were induced by 0.1 mM KNO_3_ in S but not in M. The two identified glutamate dehydrogenase genes, *PpGDH1* and *PpGDH2*, had different expression patterns in the two rootstocks. The expression level of *PpGDH1* in S was higher than that in M, and its expression was not affected by KNO_3_. In contrast, the expression level of *PpGDH2* in S was lower than that in M, and its expression was inhibited by KNO_3_ (Figure 5A). Zheng et al. have found that there is a negative feedback loop between *NRT2.1* expression and ethylene biosynthesis and signaling under nitrogen deficiency [20]. Here, we analyzed the expression levels of some ethylene-responsive genes in the transcriptome data, and the results showed that the expression levels of ethylene-responsive genes in M were higher than those in S (Figure 5B). The results showed that the expression levels of key genes in the nitrogen metabolism pathway were different in the two rootstocks, which might be the reason for the different NUE of the two rootstocks. In addition, the high expression of ethylene-responsive genes indicated that the ethylene signal was stronger in M than in S, so the expression of *PpNRT2* gene family might be affected, which would need further work to confirm.

**Figure 6:**
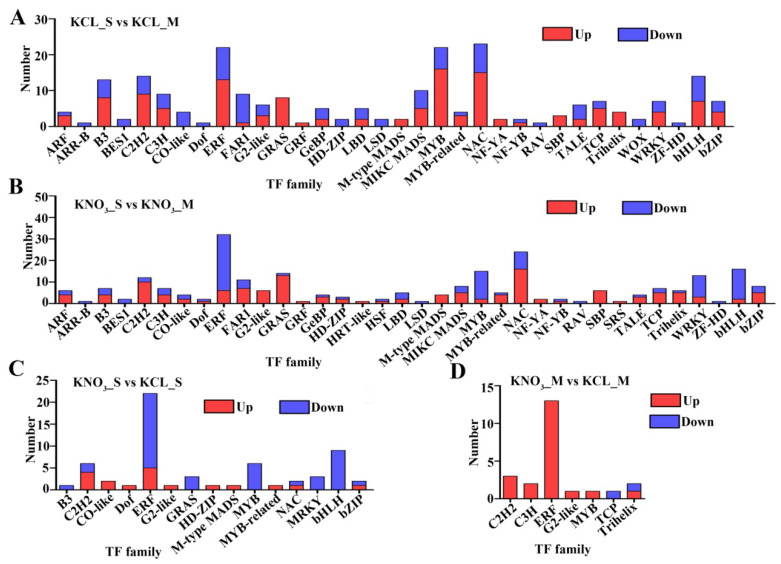
The number of differentially expressed transcription factor (TF) identified in the four comparison groups. (**A**,**B**) Transcription factor families identified in the comparison between S and M treated by KCL and KNO_3_. (**C**,**D**) Transcription factor families identified in the comparison between KCL and KNO_3_ treatment in S and M. Blue: down-regulated TFs, Red: up-regulated TFs.

Transcription factors (TF) can regulate gene expression and play an important role in nitrogen metabolism. Among the DEGs detected in this study, a total of 367 TFs were identified, belonging to 37 TF families. The TF families with the largest number of DEGs were ERF family (46 members), NAC family (35 members), MYB family (33 members) and bHLH family (25 members). After 0.1 mM KNO_3_ treatment, compared with the control group, among the DEGs between S and M, there were more members of HRT-like family, HSF family and SRS family, but less members of WOX family, and the number of down-regulated genes in ERF and MYB families was increased (Figure 6A,B). The DEGs in S that respond to KNO_3_ included members of B3 family, Dof family, HD-ZIP family, NAC family and bHLH family, but not in M. At the same time, we also found that after KNO_3_ treatment, most members of ERF and MYB families and some members of C2H2 family were down-regulated in S, but up-regulated in M. Some members of TCP family were down-regulated in M, but no members of TCP family belonged to DEGs in S (Figure 6C,D). The results indicated that these TFs were involved in nitrogen metabolism, and their different expression patterns in the two rootstocks might be another reason for the difference in NUE.

**Figure 7:**
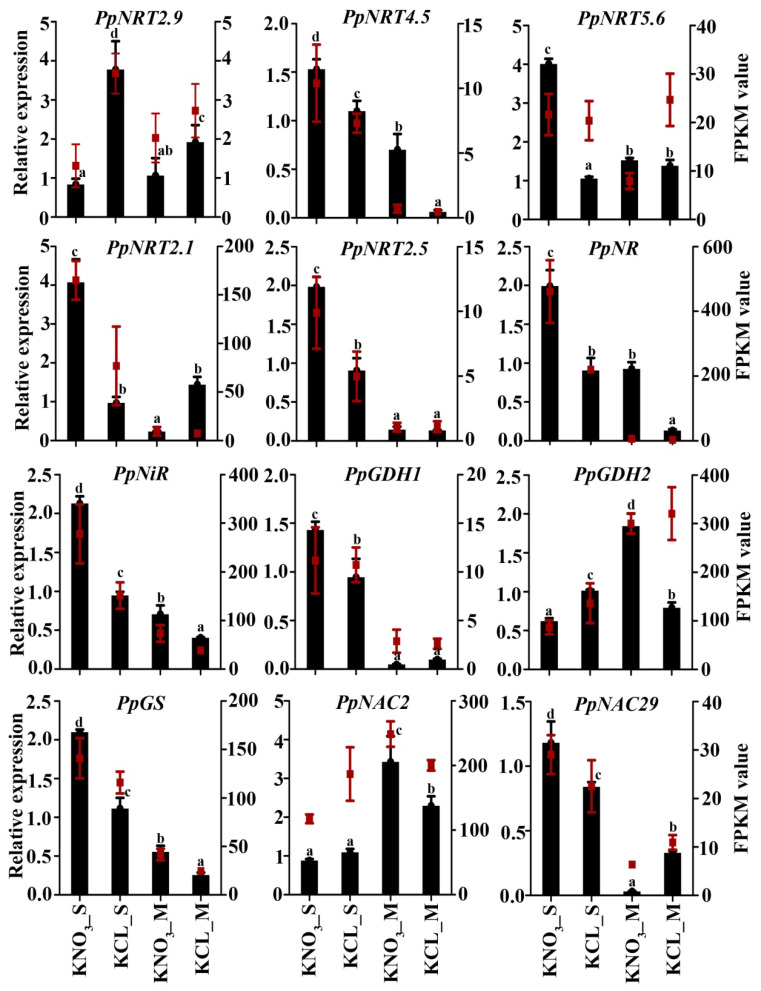
qRT-PCR validation of the KNO_3_ induced fold changes detected in S and M using RNA-seq. The relative expression values determined by qRT-PCR are presented as black columns with the left Y axis as reference. The FPKM value of DEGs are represented as red scatters with the right Y axis as reference. Different lowercase letters indicate significant differences at *p* < 0.05 by one-way ANOVA with Duncan’s (D) multiple comparisons test for the relative expression values determined by qRT-PCR. Error bars show the standard error between three biological replicates (*n* = 3).

To confirm the expression difference of the DEGs, we analyzed the expression of 12 DEGs using qRT-PCR. The expression level of each gene in S and M under KNO_3_ treatment and control was compared with its abundance from the sequencing data of the cDNA library. Results showed that among the 12 genes, except for *PpNRT5.6* and *PpGDH2* in M, the other 10 genes had the same expression pattern in S and M under KNO_3_ treatment and control as that from sequencing data (Figure 7).

### 2.5. Phenotypic Characterization of OE-PpNRT2.1 Arabidopsis

**Figure 8:**
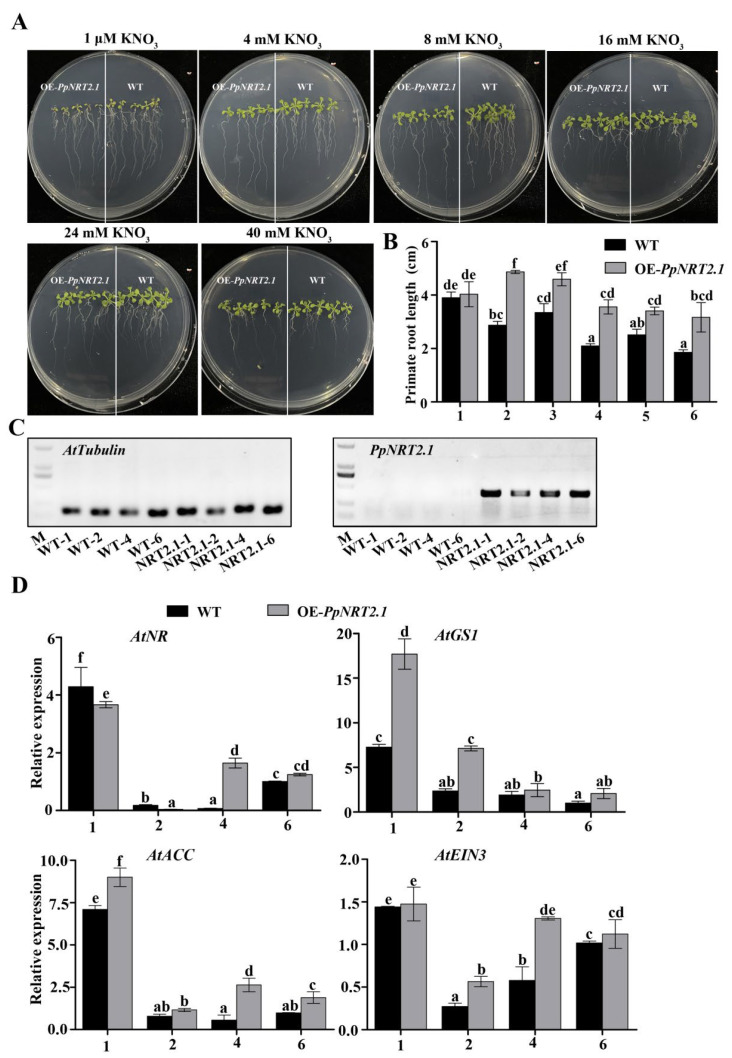
Root architectural changes of different genotype Arabidopsis seedlings treated with different concentrations of KNO_3_. (**A**) Image of 10 d -old wild type and transgenic Arabidopsis seedlings (OE-*PpNRT2.1*#2). The diameter of the petri dish is 9 cm. (**B**) The primary roots response to applied KNO_3_. Each value is expressed as mean ± SD. Error bars show the standard error between primary root length of 5 seedlings (*n* = 5 seedlings). 1–6 represents the six groups in (A), each group is treated by a concentration of KNO_3_, and the concentration of KNO3 is from 1 μM to 40 mM. (**C**) PCR analysis for wild type and transgenic Arabidopsis used cDNA as templates. M indicates DNA marker. The bands from top to bottom in Marker represent 2000 bp, 1500 bp, 1000 bp, 750 bp, 500 bp, 250 bp and 100 bp, respectively. bp: base pair. 1, 2, 4 and 6 represent wild type (WT) and transgenic Arabidopsis (*PpNRT2.1*) seedlings treated with 1 μM KNO_3_, 4 mM KNO_3_, 16 mM KNO_3_ and 40 mM KNO_3_. The electrophoretic image on the left is the PCR analysis of *AtTubulin*, and the electrophoretic image on the right is the PCR analysis of *PpNRT2.1*. (**D**) Relative expression of *AtNR*, *AtGS1*, *AtACC* and *AtEIN3* in roots of Arabidopsis seedlings with different genotypes. 1, 2, 4 and 6 represent wild type and transgenic Arabidopsis seedlings treated with 1 μM KNO_3_, 4 mM KNO_3_ 16 mM KNO_3_ and 40 mM KNO_3_. Different lowercase letters indicate significant differences at *p* < 0.05 by one-way ANOVA with Duncan (D)’s multiple comparisons test. Error bars show the standard error between three biological replicates (*n* = 3).

In Arabidopsis, rice and other plants, the role of *NRT2.1* has been reported to be involved in nitrate absorption and transport and improve the NUE of plants, but its role in fruit trees like peach, has not been reported. A heterologous gene expression assay can reflect the role of a gene in native plant to a certain extent. *PpNRT2.1*, which was one of DEGs in nitrogen metabolism in the four library comparison groups, was selected and expressed in Arabidopsis. OE-*PpNRT2.1* lines and wild-type Arabidopsis (WT) were treated with different concentrations of KNO_3_. It could be seen that high concentrations of nitrate inhibited the growth of Arabidopsis primate roots, and the primate root lengths of transgenic lines were 3.32% (1 μM), 69.49% (4 mM), 37.11% (8 mM), 69,65% (16 mM), 35.51% (24 mM) and 76.13% (40 mM) longer than those of WT in each concentration treatment group, and the differences were significant, but the lateral root growth of the transgenic lines was inhibited (Figure 8A,B). By qRT-PCR, we also analyzed the expression levels of *AtNR*, *AtGS1*, *AtACC* and *AtEIN3* in Arabidopsis roots of different genotypes under 4 concentrations of KNO_3_. In 1 μM (1) and 4 mM (2) KNO_3_ treatment groups, the expression of *AtNR* in transgenic plants was 12.86% and 77.62% lower than that of WT, and in 16 mM (4) and 40 mM (6) KNO_3_ treatment groups, the expression of *AtNR* gene in transgenic plants was 25 times and 23.57% higher than that in WT. In the 4 concentrations of KNO_3_, the expression levels of *AtGS1*, *AtACC* and *AtEIN3* genes in transgenic plants were higher than those in WT. For example, in 4 mM (2) KNO_3_ treatment group, the expression levels of *AtGS1*, *AtACC* and *AtEIN3* in transgenic plants were 205.11%, 50.26% and 109.64% were higher than those in WT (Figure 8C,D). 

**Figure 9:**
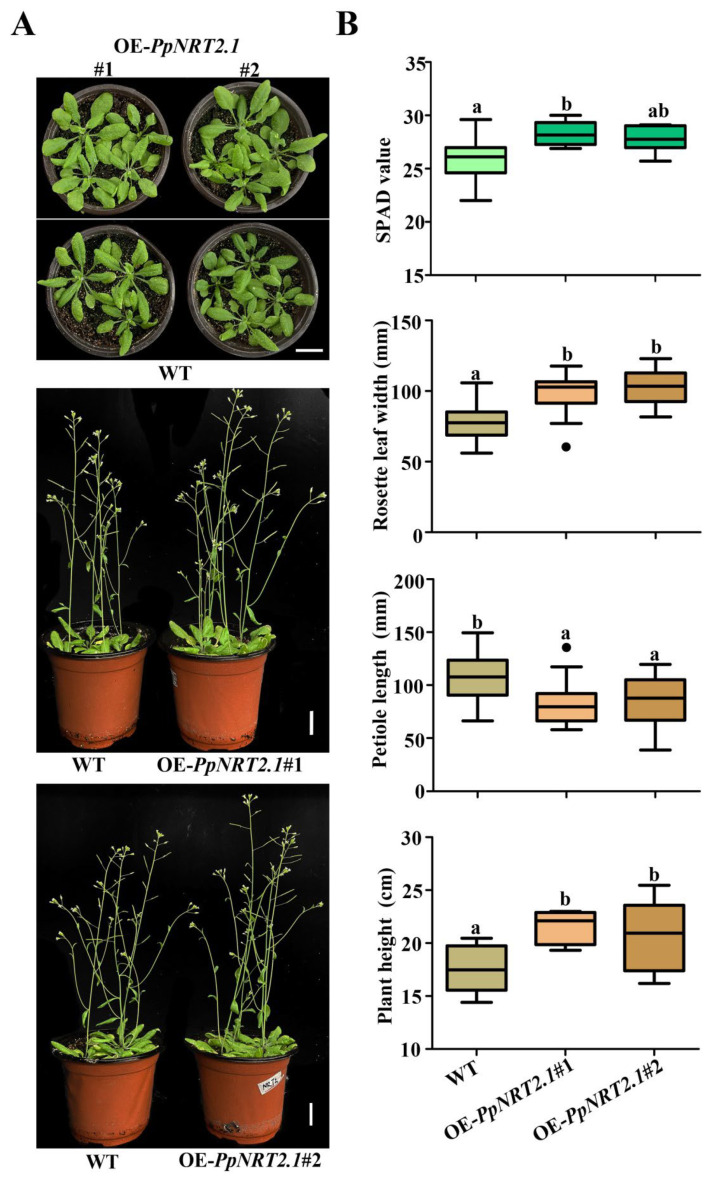
Morphology of Arabidopsis plants with different genotype. (**A**) Photographs of representative plants growing in artificial vegetative soil. Scale bar: 2 cm. (**B**) Comparison of OE-*PpNRT2.1* and WT leaf SPAD, leaf width, petiole length and plant height. Black dots represent outliers. At least 5 statistical values of the indicators for each genotype (*n* ≥ 5). Different lowercase letters indicate significant differences at *p* < 0.05 by one-way ANOVA with Duncan (D)’s multiple comparisons test.

We also analyzed the plant height and leaf SPAD of different genotypes of Arabidopsis. These indicators of transgenic plants were 13.90% and 8.39% higher than those of WT, and the differences were significant (Figure 9). The phenotype was similar to that of OE-*NRT2.1* plants that have been reported in *Arabidopsis thaliana* [14,15]. In addition, we also found that the rosette leaves of transgenic Arabidopsis became 27.85% wider and petioles were 24.36% shorter than those of WT, the differences were significant (Figure 9B). The results suggested that *PpNRT2.1* was involved in the nitrate metabolism of peach.

## 3. Discussion

In the production of peach, fruit farmers unilaterally pursue yield, so that excessive nitrogen application has become a universal phenomenon. Excessive application of nitrogen not only reduces peach fruit quality and causes tress physiological diseases, but also causes serious environmental pollution. Many fruit trees, such as apple, pear and citrus, rootstocks with high NUE have been screened [26,27], but no study is done at molecular level to explore the reasons for high NUE. The expression of genes determines the phenotype of plants, so studying the nitrogen utilization mechanism of fruit trees can help people fundamentally understand the nitrogen utilization rules of plants and formulate more efficient fertilization and breeding measures.

In this study, by measuring NUE and indicators related to NUE of two peach rootstocks, Shannong–1 and Maotao, it was found that NUE of the two rootstocks was different, and NUE of S was significantly higher than that of M (Figure 1A). Nitrate enters cell from outside through carriers, and then causes a series of metabolic changes inside of the cell. Using high-throughput sequencing technology, we obtained many DEGs from the two rootstocks under low concentration of KNO_3_ treatment. Through principal component analysis of the high-throughput sequencing results, it was showed that S was more sensitive to low concentration of KNO_3_ than M (Figure 1B), and GO and KEGG analyses showed that these identified DEGs were involved in multiple biological pathways, including secondary metabolites biosynthesis, starch and sucrose metabolism, biosynthesis and response of plant hormones, amino acid metabolism, nitrate metabolism, etc. (Figure 3 and Figure 4), which was similar to the RNA-sequencing of potato [28]. The results preliminarily explained the difference in NUE between the two rootstocks at the molecular level. This study explored many candidate genes involved in nitrogen metabolism of peach. Therefore, our work represents a transcriptome study of agronomic traits related to fruit industry, and help people understand the nitrogen metabolism of peach.

There are two types of NO_3_^−^-N transport systems in plants [11], and the high-affinity transport system relies on NRT2 protein family to transport NO_3_^−^-N when the concentration of external NO_3_^−^-N is low (NO_3_^−^-N < 1 mM) [29,30,31], and the NRT2.1 protein is the main carrier of nitrate uptake in the root maturation zone [16]. In this study, we treated rootstocks with 0.1 mM KNO_3_, and found the expression levels of *PpNRT2.1*, and *PpNRT2.5* were higher in S than in M, and their expression was induced by KNO_3_ in S, but not in M. Meanwhile, the expression levels of *PpNR*, *PpNiR*, *PpGDH1* and *PpGS* genes in S were higher than those in M (Figure 7), which was the reflection of the nitrogen assimilation pathway activated by the increase of the nitrogen inside of the cell. Moreover, under low concentration of nitrogen condition, NRT2.1 is also involved in root morphogenesis [14,15,29]. After heterologous expression of *PpNRT2.1* in Arabidopsis, the root morphology of the transgenic lines was consistent with the studies, in which the primary root length was increased and lateral root growth was inhibited (Figure 8A,B). Meanwhile, the increased expression of *AtNR* and *AtGS1* in transgenic Arabidopsis indicated the function of *PpNRT2.1* in nitrogen assimilation (Figure 8C,D). In 1 μM and 4 mM KNO_3_ treatment groups, the expression of *AtNR* was lower than that in WT, which might be regulated by other factors and need more experiments to explore.

In addition, the expression of *AtACC* and *AtEIN3* was higher in transgenic Arabidopsis roots than that in WT roots, and the expression levels were highest under low concentration of KNO_3_ treatment (Figure 8C,D), indicating that *PpNRT2.1* was involved in ethylene synthesis and signal transduction or when the expression of *NRT2.1* reached to a certain level, ethylene synthesis and signal transduction pathway could be activated. Combined with previous studies [19,20,32], which was again demonstrated that the peach *PpNRT2.1* played a role in the process of nitrogen absorption. From these above results, we speculated that one reason for the high NUE of S was the high expression of *PpNRT2.1*.

There is a negative feedback loop between *NRT2.1* expression and ethylene biosynthesis and signaling under nitrogen deficiency [20]. After KNO_3_ treatment, most of the DEGs belonging to ERF family were down-regulated in S, while up-regulated in M (Figure 6C,D). The reason for the results might be that the sensitivity of S to ethylene signal was lower than that of M. When the expression of *PpNRT2.1* increased and ethylene synthesis pathway was activated, the same concentration of ethylene could inhibit the expression of *PpNRT2.1* in M but not in S. Or in S and M, the pathway of ethylene synthesis and transduction was different. More works are needed to explore which speculate is right.

The low-affinity transport system relies on the NRT1 protein family to transport NO_3_^−^-N when the concentration of external NO_3_^−^-N is high (NO_3_^−^_-_N > 1 mM) [11,33,34]. In this study, we also identified some members of the *NRT1* gene family. Using the FPKM value as the expression level, we found that FPKM value of some members did not differ significantly between varieties and treatments, such as *PpNRT5.10*, *PpNRT8.1*, etc.. Some were significantly different between rootstocks but not respond to KNO_3_, such as *PpNRT4.6*, *PpNR5.1*, and some were not affected by KNO_3_ in S, but were affected by KNO_3_ in M, such as *PpNRT7.3*, *PpNRT5.9*, *PpNRT6.1*, etc. (Figure S3 in Supplemental Materials). The expression of these genes was not only affected by the genetic background, but also by the NO_3_^−^-N concentration. In fruit production, the application amount of nitrogen fertilizer belongs to the high NO_3_^−^-N range. Although it was difficult to speculate function of these *NRT1* family genes in the nitrate transport from this study, they might play a role in high nitrate concentration, which needs more works to explore.

Multiple transcription factors have been identified to be involved in nitrate signaling to date, including LBD gene family (*AtLBD37*, *AtLBD38* and *AtLBD39*) [35], *AtAFB3* and its target *AtNAC2* [36], *AtTGA1* and *AtTGA4* [37], *TaNAC2-5A* in wheat [21] and *OsNAC42* in rice [22]. In this study, we identified many TFs in DEGs, such as Dof family, NAC family, and HD-ZIP family, etc., and their expression levels in the two rootstocks were significantly different, and their responses to KNO_3_ were also different (Figure 6). In peach, the nitrogen metabolism mechanism is not understood well, and the regulatory role of these TFs in the nitrogen signaling in peach is still unclear, but which provides more candidate genes for us to study the mechanism of nitrogen signaling in peach.

## 4. Materials and Methods

### 4.1. Materials for Determination of NUE

The work was carried out at the experimental base of Shandong Agricultural University on 10 April 2020 (Tai’an, China). When Shannong–1 cuttings and Maotao seedlings had grown for one month, we took the whole plants out of the soil, truncated the primate roots and longer lateral roots of Maotao and adventitious roots of Shannong–1, and then planted them in the same field. A year later, the roots of the selected plants were slightly pruned again to ensure the consistent root architecture before being transplanted into pots. The 1-year-old Maotao (*Prunus davidiana* Franch) seedlings and the 1-year-old Shannong–1 cutting seedlings with the same growth potential were selected and transplanted into pots with 52 cm in outer diameter and 33 cm in height, with one plant per pot and 20 plants of each rootstock. The soil for potted plants was collected from the surface garden soil of 0–25 cm and was naturally air-dried and sieved to remove impurities. The basic physical and chemical properties of the tested soil: pH 6.77, the content of alkaline hydrolyzable nitrogen was 57.18 mg/kg, the content of organic matter was 11.13 g/kg, the content of available phosphorus was 55.27 mg/kg, and the content of available potassium was 78.45 mg/kg. The seedlings were managed normally after transplanting. On 22 August, fertilization was carried out, and 3.5 g urea (0.5 g of which was marked with ^15^N) was applied to each seedling. ^15^N urea (^15^N = 10.08%) was provided by Shanghai Engineering and Technology Research Center for Stable Isotops, Shanghai China. Common urea was purchased from Aladdin, Shanghai, China. The SPAD and net photosynthetic rate of leaves were measured on 31 August. After one month of treatment, three plants of each peach rootstock were randomly selected, crushed, and sampled. The SPAD and net photosynthetic rate were measured by Spad-502 Plus chlorophyll analyzer (Konica Minolta, Inc., Tokyo, Japan) and portable photosynthesis meter with CIRAS-3 (PP Systems, Amesbury, MA, USA), respectively.

### 4.2. Determination of Root Vigor

For the determination of root vigor, nine trees of each rootstock were selected and each of the three trees was used as a biological replicate. The root system of the whole plant was washed out from the soil, and the white young roots without lignification were cut and washed five times with distilled water. Then 0.5 g root tips of each rootstock were selected for determination of root vigor. The root vigor was determined using Root Vitality Detection kit (TTC method) (Shanghai Lianmai Biological Engineering Co., Ltd., Shanghai, China).

### 4.3. Measurement of NUE

After one month of treatment, three plants of each peach rootstock were randomly selected. The leaves, shoots, trunk, thick roots (diameter > 2 mm), and fine roots (diameter < 2 mm) of the whole plant were sorted and collected respectively. The samples of each part were cleaned successively with clean water, detergent, clean water, 1% hydrochloric acid and 3 times distilled water. After removing the distilled water on the surface with absorbent paper, the samples were placed in a drying oven (Shanghai Yiheng Scientific Instrument Co., LTD, Shanghai, China) at 105 °C for 30 min, and then dried at 75 °C to constant weight. The samples were crushed with a stainless steel mill and passed through an 80-mesh sieve. The ^15^N was measured by mass spectrometer (Finnigan, CA, USA), and the total nitrogen content was measured by automatic kieldahl apparatus (Hanon, Dalian, China). The NUE of rootstocks was determined as described by Zhang et al. [38].

### 4.4. Materials for the Construction of cDNA Libraries

The perennial peach rootstock Shannong–1 was planted in the experimental base of Shandong Agricultural University (Tai’an, China), and the lignified shoots were collected for peach cuttings, and the work was carried out in a greenhouse of experimental base of Shandong Agricultural University, on 10 September 2021. The lignified shoots were cut into segments with 4–5 buds and one leaf. The upper end of the cuttings was cut flat, and the lower end were cut a 2 cm long bevel with a scalpel. Soak the lower end of the cuttings in 1 g/L IBA (Solarbio, Beijing, China) solution for 10 min, then take out and insert them into the river sand obliquely. The river sand was watered thoroughly and kept moist by spraying during the whole experiment. After one month, the rooted cuttings were removed from the river sand, and together with the one-month-old Maotao seedlings transferred to washed quartz sand (Shandong Jinan Quartz Sand Co. LTD, Jinan, China), which was kept moist with double distilled water. The primate roots and longer lateral roots of Maotao and adventitious roots of Shannong–1 were truncated before the plants being transferred to washed quartz sand. After one week, the two rootstock seedlings were treated with 0.1 mM KNO_3_ (Sinopharm Chemical Reagent Co. LTD, Shanghai, China), and the control group was treated with 0.1 mM KCL (Sinopharm Chemical Reagent Co. LTD, Shanghai, China). The white young roots were collected after 0.5 h, frozen in liquid nitrogen and stored in an ultra-low temperature freezer (Haier, Qingdai, China) for high-throughput sequencing and subsequent experiments. Three biological replicates were made for each treatment, with 15 seedlings per rootstock per replicate.

### 4.5. RNA Extraction and cDNA Synthesis

Total RNA was isolated from roots using a modified CTAB method [39] and treated with DNase I (Invitrogen, Carlsbad, CA, USA) to remove DNA contamination. RNA integrity was verified by electrophoresis on a 1.2% agar gel, and the concentration was quantified using a NanoDrop 1000 (Thermo, Waltham, MA, USA). The RNA was used for RNA sequencing and real-time PCR. Approximately 2 μg of RNA was used as template for first-strand cDNA synthesis using SuperScript reverse transcriptase (Invitrogen, CA, USA) for analyzing the abundance of mRNAs. Total RNA from each of the three biological replicates was independently used in qRT-PCR analysis.

### 4.6. RNA Sequence Analysis

Total RNA was extracted from the roots of Shannong–1 and Maotao treated by 0.1 mM KNO_3_ and 0.1 mM KCL for 0.5 h. RNA-seq libraries were prepare according to the Illumina Standard library preparation kit. Illumina HiSeq 2500 was as a platform for RNA-seq at Qingdao OE Biotech. RseqQC was used for quality control checks on raw sequencing data. Clean reads were aligned to the peach reference genome (ftp://ftp.ncbi.nlm.nih.gov/genomes/all/GCF/000/346/465/GCF_000346465.2_Prunus_persica, accessed on 2 February 2017) using Hisat. The gene expression value was normalized as FPKM. A gene was considered to be expressed only when the FPKM value was greater than zero in the three biological replicates. Use DESeq2 software [40] to standardize the number of counts of each gene (use BaseMean value to estimate the expression level), calculate the fold of difference, and use NB (negative binomial distribution test) to test the significance of difference, and finally screen differential protein-coding genes according to the fold of difference and difference significance test results. The default criteria for screening differences were q < 0.05 and fold change > 2 or < −2.

### 4.7. Gene Functional Annotation

The Gene Ontology (GO) database (http://www.geneontology.org/, accessed on 1 April 2005) was searched to annotate the putative genes involved in cellular components, biological processes, and molecular functions, and the GO terms of biological processes was analyzed using R package. All DEGs were mapped to GO terms in the GO database by counting the percentage of gene numbers for each term. We used hypergeometric distribution text to calculate the *p* value. GO terms with a *p* value ≤ 0.05 were considered to be significantly enriched in DEGs.

Pathway analysis was performed on differential protein-coding genes using the KEGG database (combined with KEGG annotation results) (http://www.genome.jp/kegg/, accessed on 7 November 2019), and the significance of DEGs enrichment in each pathway term was calculated using the hypergeometric distribution test.

### 4.8. Quantitative RT-PCR (qRT-PCR) Analysis

qRT-PCR was conducted in 10-μL reactions using SYBR Green Supermix (TaKaRa, Kusatsu, Japan) to analyze the abundance of DEGs. The primers used for these reactions were listed in Table S2 in Supplementary Materials. The relative abundance of mRNAs was calculated by the 2^-ΔΔCT^ method and normalized by *PpTEF2* and *Atactin11* as references for peach and Arabidopsis, respectively [20,41].

### 4.9. Plant Material and Growth Conditions

*Arabidopsis thaliana* Columbia (Col) was used as the wild type. Seeds were sown on a mixture of vermiculite and nutritive soil, and the plants were maintained at 21 °C with a 16 h light and 8 h dark photoperiod. For root phenotype analysis, Arabidopsis seeds (WT and OE-*PpNRT2.1*) were sterilized with 75% alcohol (Tianjin Kaitong Chemical Reagent Co. LTD, Tianjin, China) for 30 s and 2% sodium hypochlorite (Tianjin Kaitong Chemical Reagent Co. LTD, Tianjin, China) for 8 min, and then washed 5–6 times with sterile water. The seeds were sown on medium containing different concentrations of KNO_3_. The culture dish was placed vertically, and the growth of the seedlings was observed regularly and photographed.

### 4.10. Construction of Plant Expression Vector, Arabidopsis Transformation and Characterization of Transgenic Plants

The *PpNRT2.1* coding sequence was amplified using the primers listed in Table S2 in Supplementary Materials and cloned into the pCambia1300 vector under the control of CaMV 35S promoter. The construct was confirmed by DNA sequencing before being introduced into the *Agrobacterium tumefaciens* strain GV3101. The floral dipping method was used to create transgenic plants [42]. Transgenic seeds were screened on MS (Murashige and Skoog) (Hopebio, Qingdao, China) plated containing 30 mg/L hygromcin (GENVIEW, Tallahassee, USA), and the transgenic lines were identified with following primers: 5′-TCATATCGGGCCTCACTGGA-3′ and 5′-ACCCCGGTGAACAGCTCCTC-3′. Homozygous T3 seeds of 4 representative lines were used for phenotypic analysis. Primers were synthesized by Sangon Bioengneering (Shanghai) Co. LTD, Shanghai, China.

### 4.11. Statistical Analysis

All data in this study were obtained from at least three independent experiments. Data were plotted as means ± SD, and error bars were standard deviation. The data were analyzed using GraphPad Prism 5.01 software, and significant differences were analyzed with Student’s *t* test and one-way ANOVA with Duncan’s (D) multiple comparisons test using IBM SPSS Statistics 20 software.

## 5. Conclusions

**Figure 10:**
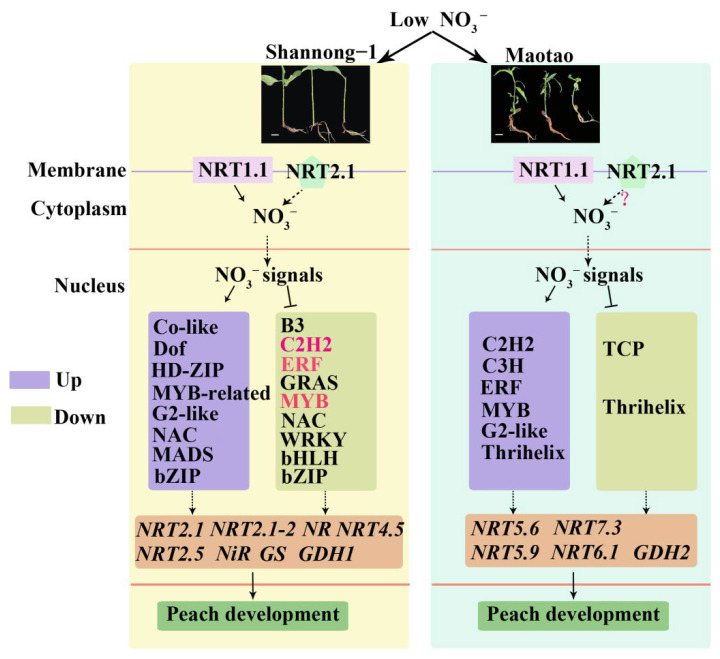
Model diagram of effects of low NO_3_^−^ on Shannong–1(S) and Maotao (M). Depicted on the left is the pathway involved in nitrogen metabolism in S, and depicted on the right is the pathway involved in nitrogen metabolism in M. Under low-concentration KNO_3_ treatment, the NRT2.1 protein in S participated in the transport of NO_3_^–^ in a certain way. After NO_3_^–^ enter the cell of S, it promoted the expression of multiple transcription factors such as co-like and NAC, and also inhibited the expression of some transcription factors such as B3, C2H2. Changes in the expression level of these transcription factors increased the expression of multiple genes in the nitrogen metabolism pathway, including *PpNRT2.1*, *PpNR*, *PpGS*, etc. In M, low-concentration KNO_3_ treatment did not make the expression of *PpNRT2.1* change, so it was uncertain whether NRT2.1 protein is involved in the transport of NO_3_^–^. After NO_3_^–^ enter the cell of M, it promoted the expression of some transcription factors such as C2H2 and ERF, while inhibited the expression of TCP and Thrihelix transcription factors. Changes in the expression of these transcription factors made the expression of *PpNRT5.6*, *PpNRT5.9*, *PpGDH2* and other genes change. Arrow: positively, T-bar: negatively, dashed arrow: regulation by some means.

In this study, through high-throughput sequencing, we explored the molecular mechanism of nitrogen metabolism in peach rootstocks for the first time and explored some internal regulatory factors (especially transcription factors) that regulate the NUE of peach trees (Figure 10). Shannong–1 and Maotao had different responses to 0.1 mM KNO_3_, and the expression of genes was also different. There were more transcription factor families with changes in expression in Shannong–1 than in Maotao, and the expression patterns of some transcription factors in the two rootstocks were opposite, especially the ERF transcription factor family. In Shannong–1, the expression of genes encoding nitrogen reductase on the nitrogen metabolism pathway and NRT2 family members responsible for the high-affinity transport system was induced, but not in Maotao. This result explained the difference in the NUE of the two rootstocks at the molecular level. The study provided genetic reserves for the molecular breeding of peach with high NUE and a theoretical basis for people to formulate more reasonable fertilization methods.

## Data Availability

Data is contained in Supplementary Materials.

## Supplementary Materials

The following supporting information can be downloaded at: https://www.mdpi.com/article/10.3390/ijms231911144/s1.

## Abbreviations

S: Shannong–1; M: Maotao; NUE: Nitrogen Use Efficiency; SPAD: Soil and Plant Analyzer Development; *P*_N_: Net photosynthetic rate; OE: Over expression; SD: Standard Deviation. TF: Transcription factor; GO: Gene ontology; KEGG: Kyoto Encyclopedia of Genes and Genomes; HATS: High affinity transport system; LATS: Low affinity transport system; NRT: Nitrate transporter.
