# Supplementary material for: Comparative Transcriptome Analysis of Two Peach Rootstocks Uncovers the Effect of Gene Differential Expression on Nitrogen Use Efficiency
Source: Int J Mol Sci. 2022 Sep 22;23(19):11144. doi: 10.3390/ijms231911144 (PMC9570093; doi:10.3390/ijms231911144)
Supplement: Supplementary file 1 [file ijms-23-11144-s001.zip › ijms-1897150-Figures S1-S3.pdf]

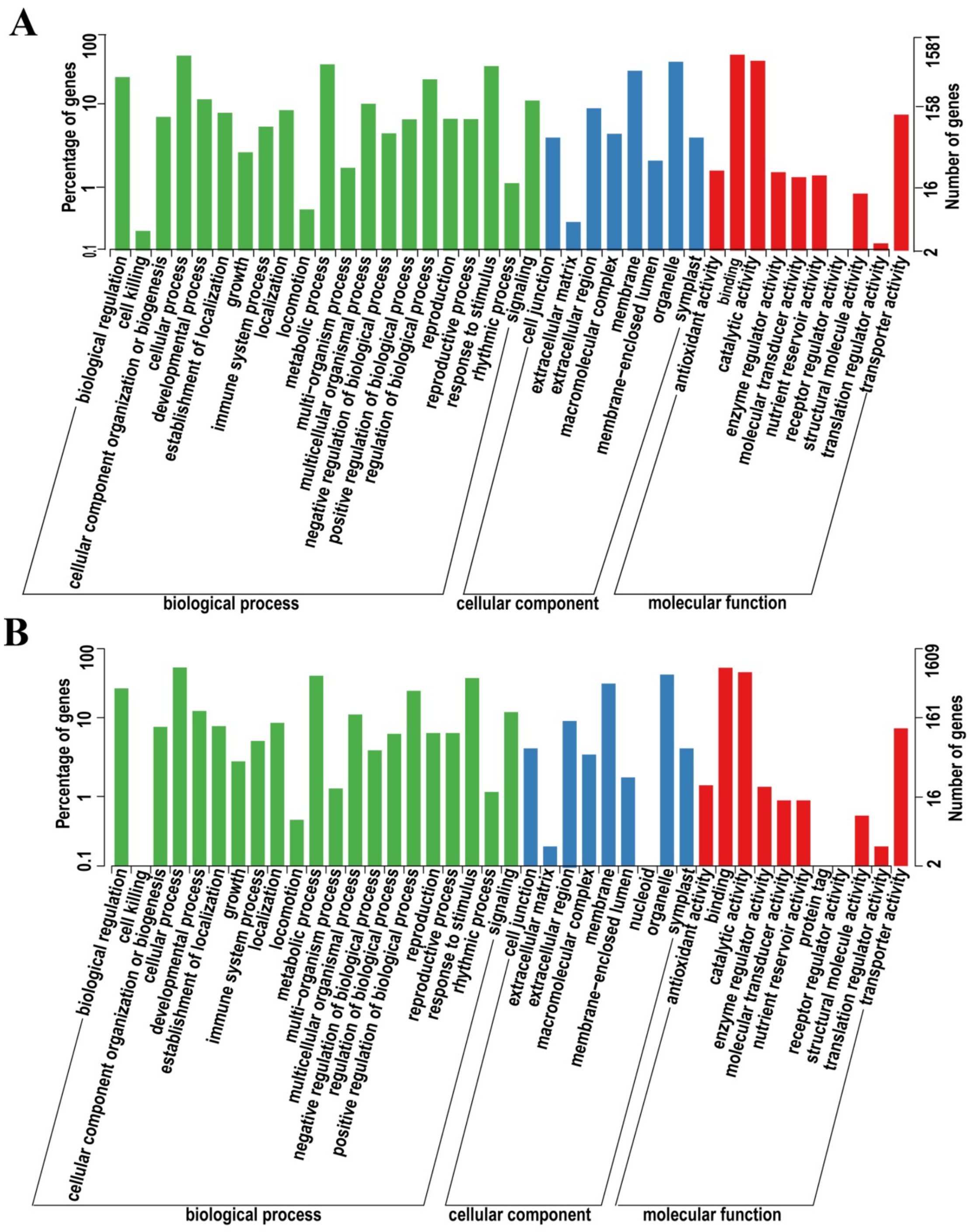

Figure S1. Gene ontology categories of the DEGs between the S and M. (**A**) Gene ontology categories of DEGs between S and M treated by 0.1 mM KCL. (**B**) Gene ontology categories of DEGs between S and M treated by 0.1 mM KNO_3.

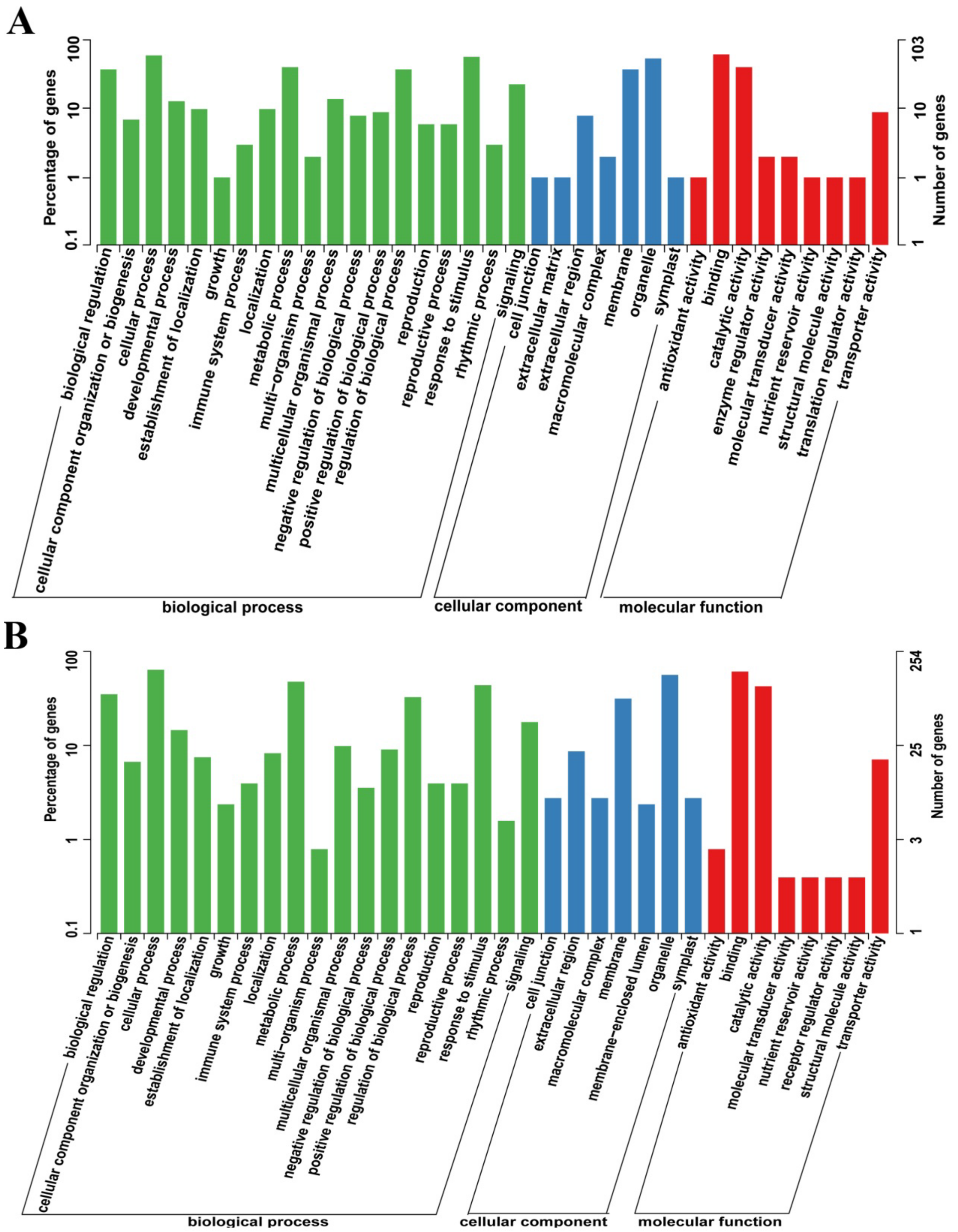

Figure S2. Gene ontology categories of the DEGs between different treatments of the same rootstock. (**A**) Gene ontology categories of DEGs between KNO_3 treatment group and control group of M. (**B**) Gene ontology categories of DEGs between KNO_3 treatment group and control group of S.

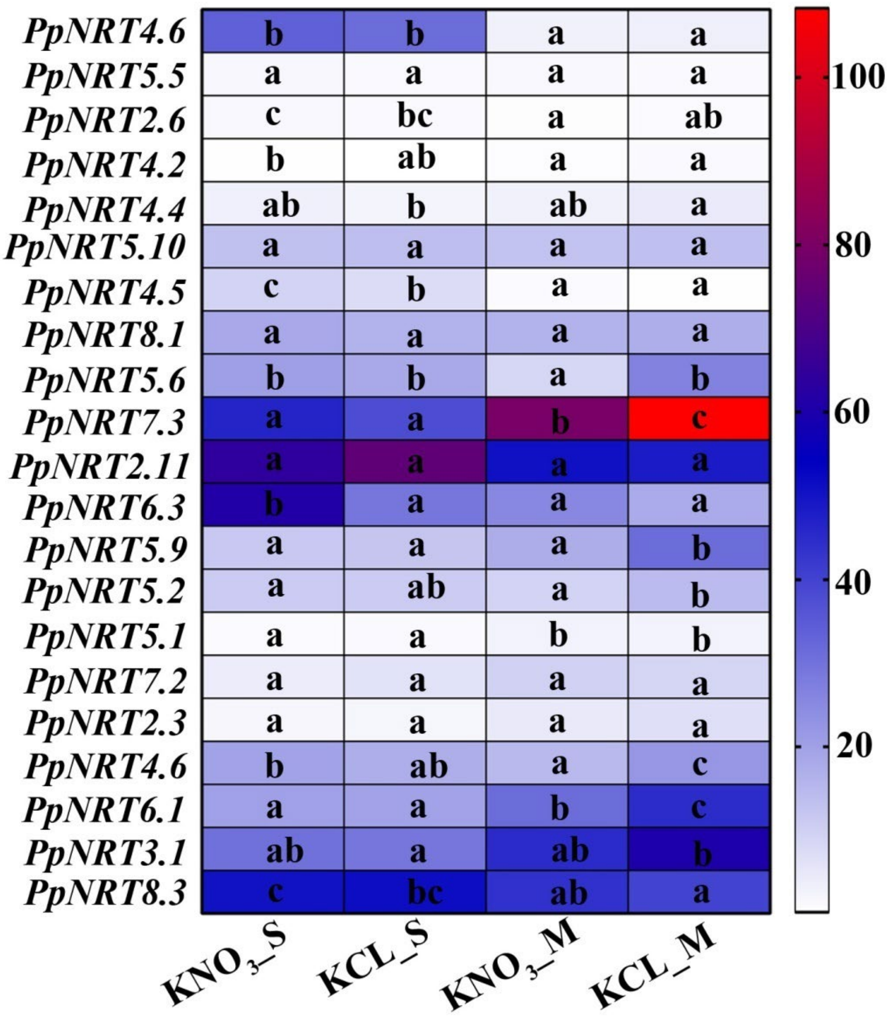

Figure S3. The normalized expression level (FPKM) of some differentially expressed *PpNRT1* gene family members in four libraries analyzed by heat map. Different lowercase letters indicate significant differences at $p < 0.05$ by one-way ANOVA with Duncan(D)'s multiple comparisons test.
