# Supplementary material for: Comparative Transcriptome Analysis of Two Peach Rootstocks Uncovers the Effect of Gene Differential Expression on Nitrogen Use Efficiency
Source: Int J Mol Sci. 2022 Sep 22;23(19):11144. doi: 10.3390/ijms231911144 (PMC9570093; doi:10.3390/ijms231911144)
Supplement: Supplementary file 1 [file ijms-23-11144-s001.zip › ijms-1897150-Table S2.pdf]

Table S2. Primers used in the study.

Primers for qRT-PCR		
**Primers Name**	**Forward Primer (5'–3')**	**Reverse Sequence (5'–3')**
PpNRT2.9	TGGGTTTTTCTTCATTCGCTTCTTTTTT	GTTGTTGGCCCTTTACATGTTTCTCCTT
PpNRT4.5	TATACGCCACGGAGGGTCTT	GGGTGGTAGCCGACTTTGTC
PpNRT5.6	ATCACATCACGGAGAAGGGTGGGAAG	TGTAAGAGTAACGCCGAGCCAAGAAAAC
PpNRT21.	TCATATCGGGCCTCACTGGA	GCACACGATCATTACCCCCA
PpNRT2.5	AGCGTTTCACTTGTCCTGGT	CAAGCCGCGGTCCAAATAAG
PpNR	TCGTCCGCTCCTTCAACTTC	TTAAATCCCCGTACTCGCCG
PpNiR	CCTCTTGATGCCTGGGTCTC	CTCGTCTTCTGTCGGTTCCC
PpGDH1	TCAAGGGGTTCAATGGTGGG	TCAGTGGGGTGATTAGCTGC
PpGDH2	CAAGCCGCAATTTTCGCAAC	AAGCTTCCATCGTCCTTCGG
PpGS	GCACAGATTTTGGCACCCAC	AGCAGAGCTCTTGGTTGGTC
PpNAC2	AGGGACCGGAAGTATCCGAA	CGGCGTAGAAAACCAAAGCC
PpNAC29	CAGGAATCCCAAGCACCGAA	TGTCGGACTGAATCGTTGCG
PpTEF2	GGTGTGACGATGAAGAGTGATG	TGAAGGAGAGGGAAGGTGAAAG
AtNR	CCACGTCCCTAAAGCCCAAT	TCTGTTCCTTACGGCGGTTC
AtGS1	ATGGTGAAGGCAACGAGAGG	CCTACTCTCACCGATGCTCC
AtACC	CGGCGACGTTTTCCTCATTC	GCGGCGTCAACGGTTAATTT
AtEIN3	CCTGCGAGAATCTTGGGTGT	TGCTCCATACGGTAAGCGAC
Atactin11	CCACATGCTATTCTGCGTTTGGACC	CATCCCTTACGATTTCACGCTCTGC

Primers for vector construct		
Primers name	Forward primer (5'–3')	Reverse sequence (5'–3')
1300-PpNRT2.1	ATTTGGAGAGGACAGGGTAC-<br>CATGGCCGAAGTCGAAGGTGAA	CACCATGGTACTAGTGTCGACAACATGG-<br>GAAGGTGTGGTGTT

The sequence of the restriction enzyme is underlined.
